# Adaptive radiations in natural populations of prokaryotes: innovation is key

**DOI:** 10.1093/femsec/fiad154

**Published:** 2023-11-23

**Authors:** Michiel Vos, Daniel Padfield, Christopher Quince, Rutger Vos

**Affiliations:** European Centre for Environment and Human Health, University of Exeter Medical School, Environment and Sustainability Institute, Treliever Road, Penryn Campus, Penryn, TR10 9FE, United Kingdom; Environment and Sustainability Institute, University of Exeter, Treliever Road, Penryn Campus, Penryn, TR10 9FE, United Kingdom; European Centre for Environment and Human Health, University of Exeter Medical School, Environment and Sustainability Institute, Treliever Road, Penryn Campus, Penryn, TR10 9FE, United Kingdom; Environment and Sustainability Institute, University of Exeter, Treliever Road, Penryn Campus, Penryn, TR10 9FE, United Kingdom; Organisms and Ecosystems, Earlham Institute, Norwich Research Park, Norwich NR4 7UZ, United Kingdom; Gut Microbes and Health, Quadram Institute, Norwich Research Park, Norwich NR4 7UQ, United Kingdom; Naturalis Biodiversity Center, Understanding Evolution, Darwinweg 2, Leiden 2333 CR, the Netherlands; Institute of Biology Leiden, Leiden University, Sylviusweg 72, Leiden 2333 BE, the Netherlands

**Keywords:** adaptive radiations, diversification, key innovations, macroevolution, pangenomes, speciation

## Abstract

Prokaryote diversity makes up most of the tree of life and is crucial to the functioning of the biosphere and human health. However, the patterns and mechanisms of prokaryote diversification have received relatively little attention compared to animals and plants. Adaptive radiation, the rapid diversification of an ancestor species into multiple ecologically divergent species, is a fundamental process by which macrobiological diversity is generated. Here, we discuss whether ecological opportunity could lead to similar bursts of diversification in bacteria. We explore how adaptive radiations in prokaryotes can be kickstarted by horizontally acquired key innovations allowing lineages to invade new niche space that subsequently is partitioned among diversifying specialist descendants. We discuss how novel adaptive zones are colonized and exploited after the evolution of a key innovation and whether certain types of are more prone to adaptive radiation. Radiation into niche specialists does not necessarily lead to speciation in bacteria when barriers to recombination are absent. We propose that in this scenario, niche-specific genes could accumulate within a single lineage, leading to the evolution of an open pangenome.

## Teaser

Bursts of adaptive evolution mediated by horizontally acquired key innovations could be an important driver of bacterial diversity and influence pangenome evolution.

## Introduction

**Figure 1.**
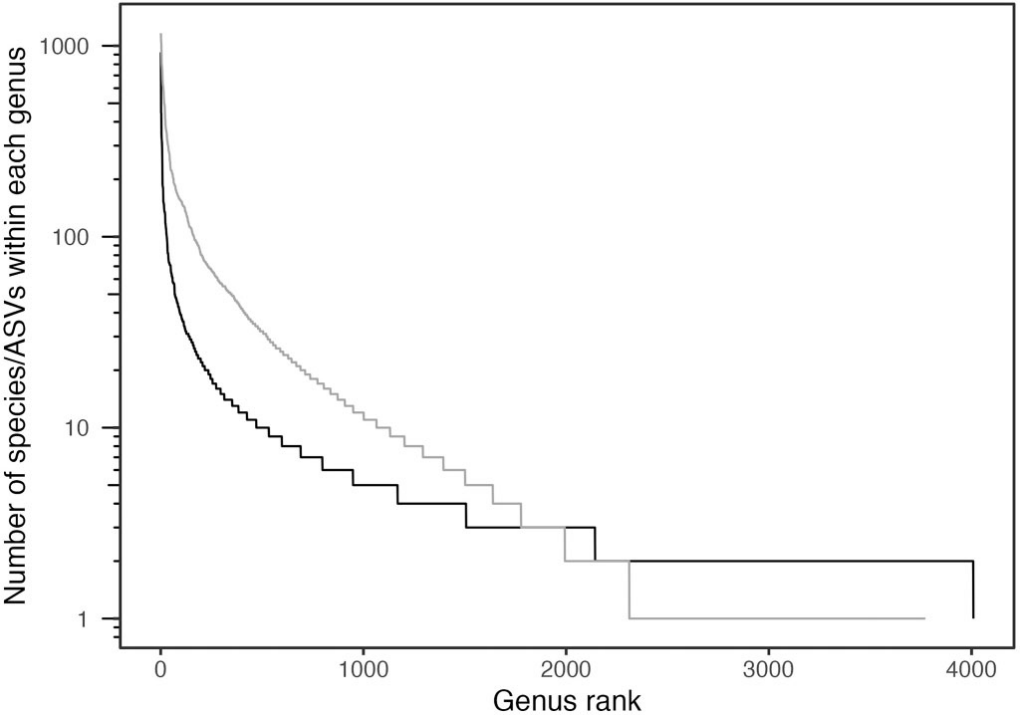
Variation in species diversity among bacterial genera. Rank abundance curves of total species diversity from all taxonomically recognized species (LPSN; https://www.bacterio.net/) (black line) and 16S rRNA-based ASVs (amplicon sequence variants) assigned to genera in the Earth Microbiome Project (grey line).

A central challenge in evolutionary biology and ecology is explaining why species richness patterns in the Tree of Life vary drastically between different taxa (Scholl and Wiens 2016, Mooers and Heard 1997). Differences in species richness are evident in many plant and animal sister clades; compare for example the lone species of Hoatzin (Order Opisthocomiformes) with the 5000+ species of passerines (Order Passeriformes). In eukaryotic taxa, such variation in species richness has long been interrogated using analyses of phylogenetic tree shape. However, whether similar heterogeneity exists in bacteria and archaea has received less attention (Dykhuizen 1998). This is partly because the study of bacterial biodiversity faces two major challenges. The first challenge is that most taxa are undersampled, hindering accurate estimates of species diversity (Quince et al. 2008) and phylogenetic reconstruction (Heath et al. 2008). As a result, estimates of total bacterial diversity vary wildly, from ∼10^4^ (Mora et al. 2011), via ∼10^6^ (Yarza et al. 2014, Louca et al. 2019), ∼10^9^ (Larsen et al. 2017) to ∼10^12^ species (Locey and Lennon 2016). Of course, estimates of species richness at least to some extent rely on how species are defined in the first place. The second challenge is that there is no one-to-one agreement between current taxonomy, species delineated based on overall genomic distance, or operational taxonomic units (OTUs) based on clustering of 16S rRNA sequences (Parks et al. 2018). Differential sampling effort and inconsistent taxonomy must mean that some of the observed intertaxon differences in bacterial species richness must be artefactual. These caveats notwithstanding, it is clear that there are substantial differences in species richness when surveying either named species or 16S amplicon-based OTUs (Fig. 1).

**Figure 2.**
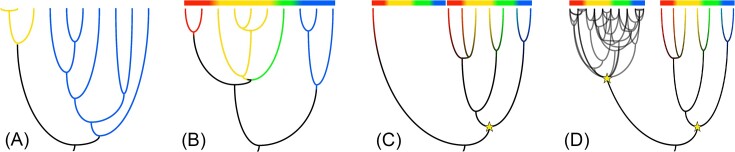
Four scenarios leading to differences in species richness between taxa. (A) All else being equal, older clades should be of larger size. The root ages for both sister clades are different, such that the blue clade has had more time to diversify than the yellow clade. (B) Clades might diversify when faced with multiple potential niches to exploit, demonstrated by partitioning and subsequent diversification into red, yellow, green, and blue niches. (C) The capacity for diversification into multiple lineages might be mediated by the presence (or absence) of a key innovation, here indicated by the star. The clade on the right has acquired the capacity to exploit multiple niches into which it diversifies, while the branch on the left does not. (D) Adaptive radiations caused by key adaptations (star symbols) in the presence of recombination barriers, allowing new niche specialists to evolve into distinct species (deep branches, right clade), or in the absence of recombination barriers, leading to the evolution of many niche specialists that do not evolve into species ‘proper’, with a shared core genome (shallow intermingled branches, left clade).

Numerous explanations for differences in species richness have been put forward but many of these, such as the effect of trophic level, body size, geographic range, latitude, or temperature (Hutchinson 1959, Rosenzweig 1995, Dykhuizen 1998), do not necessarily translate to prokaryotes (e.g. Bahram et al. 2018). However, reasoning from first principles, species richness, be it in animals, plants, or bacteria, is ultimately the product of speciation and extinction adding and subtracting species over time. Taxa with a higher net diversification rate (i.e. a higher rate of speciation than extinction) are expected to have higher species richness. However, it is possible that different clades with identical diversification rates still differ in species richness, as older clades will have had more time to accumulate new species (Fig. 2A).

Diversification can proceed at a constant rate, but can also occur in pulses (or sporadic declines). Bursts in diversification (‘rapid cladogenesis’) are commonly ascribed to the exploitation of ecological opportunity (Schluter 2000, Gavrilets and Losos 2009) (Fig. 2B). Such adaptive radiations are contingent on two main conditions: first, many niches must be available (or one large niche space that can be partitioned), and second, only few lineages must be in a starting position to fill them (i.e. competition must be relaxed). Laboratory experiments have demonstrated that frequency-dependent competition for niche space can drive adaptive radiations in bacteria. In a seminal experiment, *Pseudomonas fluorescens* predictably diversified into three types over the course of only a few days when incubated in static flasks, with wrinkly spreaders inhabiting the broth–air interface, fuzzy spreaders occupying the bottom of the flask, and the ancestral smooth morph residing in the broth (Rainey and Travisano 1998).

Phylogenetic methods offer ways to uncover bacterial diversification on much longer (geological) timescales. They often rely on PCR amplification and sequencing of the conserved 16S ribosomal marker from environmental samples serving as proxies for species or on higher-resolution concatenated core genes sequenced from isolated strains. These studies indicate that bacterial speciation rate is slightly higher than extinction rate (Loren et al. 2014, Marin et al. 2016, Louca et al. 2018) (but see Martin et al. 2004), consistent with results for multicellular organisms where turnover of taxa is high and where most diversity is now extinct (Louca et al. 2018). Some studies have uncovered bursts in diversification rate in (sections of) bacterial phylogenies (Morlon et al. 2012, O’Dwyer et al. 2015). As 16S-based datasets have limited power to detect diversification on shallower evolutionary time scales (Louca et al. 2018) and studies using higher resolution markers generally survey only a relatively limited number of taxa, such burst-like evolution could be present but overlooked in other studies.

The aim of this paper is to examine the evidence for bursts of adaptive evolution in prokaryotes and their evolutionary and ecological drivers, and how these compare to those in macroscopic species. We will discuss how differences in diversification rate between prokaryotes could affect other aspects of bacterial biology such as the evolution of pangenomes. Although highly insightful, lab experiments are generally performed on extremely short timescales that rely solely on mutation [and seldomly incorporate horizontal gene transfer (HGT), a central driver of genomic and functional diversity in bacteria] and are based on purely artificial selection pressures in the absence of other community members. We, therefore, will focus on natural populations in this review and refer to other literature summarizing results on experimental adaptive radiations in bacteria (Travisano and Rainey 2000, MacLean 2005). We will review studies on isolates assigned traditional taxonomic labels, 16S amplicons, and closely related clusters based on whole-genome sequences.

## Key innovations spur adaptive radiations in bacteria

In macrobes, the open niche space that forms a prerequisite for adaptive radiations is often provided by rare colonization events of remote localities such as mountains, lakes, or islands, where competing species are absent. Classic examples of such adaptive radiations include Darwin’s finches in the Galapagos, Cichlid fishes in East African Rift Lakes and Silversword plants in Hawaii (Schluter 2000). This scenario is not likely in bacteria, as they experience little dispersal-limitation due to their small size and high abundance, meaning niche specialists and niches will be efficiently matched. This diminished role of biogeographical barriers and allopatry in prokaryotes (and a correspondingly increased role for environmental filtering) is illustrated by many 16S-based studies (Lozupone and Knight 2005); for instance, most global soil diversity was found to be contained in an area as small as Central Park in New York City (Ramirez et al. 2014). A recent large-scale analysis of curated genomes from around the globe found that most prokaryotic clades on Earth’s surface are globally distributed (Louca 2022). Consistent with an earlier housekeeping gene-based study demonstrating geographical divergence in a thermophile archaeon (Whitaker et al. 2003), thermophiles were found to be least dispersive, which makes sense as they live in relatively small, specialized habitats that are far apart (Louca 2022). However, neither study could conclude that even extremophile species displayed endemicity. There seems to be no bacterial equivalent of marsupials, and it is ecological opportunity—rather than geographic isolation—that is most likely to drive bacterial diversification (Vos 2011). The oft-quoted adage ‘everything is everywhere, the environment selects’ thus seems to be vindicated by sequencing-based studies almost a century after it was first proposed (Baas Becking 1934).

How could adaptive radiations occur in sympatry? One pathway to ecological innovation that is not reliant on geographical isolation was developed by Miller, Mayr, and Simpson in the middle of the 20th century (Heard and Hauser 1995, Schluter 2000). These and other scientists posited that occasional evolutionary ‘key innovations’ give rise to entirely new functional capabilities that allow the colonization of new ‘adaptive zones’ (Hunter 1998, Alfaro 2014) (Fig. 2C). Such adaptations could provide a release from competition and access to niche space not available before. A well-known example in animals is the radiation of Notothenioid fishes in the Antarctic Ocean. The evolution of antifreeze glycoproteins that lower internal freezing point in their last common ancestor has allowed the invasion of comparatively empty oceanic regions with subzero temperatures and the subsequent diversification into over 130 species (Matschiner et al. 2015).

It could be argued that prokaryotes have an especially great potential to evolve key innovations, as HGT allows the wholesale acquisition of entirely novel functional traits originating from other strains and species (Lawrence 2001, Cohan and Koeppel 2008, Hall et al. 2017). One population genomics study beautifully uncovered a radiation of bacterial niche specialists driven by HGT (Hehemann et al. 2016). In previous work, the same group had identified multiple genetically distinct *Vibrio* clusters that were hypothesized to be ecologically differentiated, as they were enriched in different particle size fractions in the same seawater samples (Hunt et al. 2008). Subsequent genome sequencing uncovered that the brown algal glycan alginate pathway had undergone extensive combinatorial changes mediated by HGT within and between these clusters as well as more distantly related species, leading to rapid clade diversification. Subsequent growth rate experiments demonstrated that variation in enzyme type, copy number, and localization (on the cell wall or broadcast into the environment) translated into physiological differences, which in turn could explain the differential association of different types with particle size (representing different degradable algal cell wall types) and season (Hehemann et al. 2016). This case bears all the hallmarks of an adaptive radiation mediated via a key innovation.

Another example of an adaptive radiation driven by an HGT-acquired key innovation is offered by the Thaumarchaeota, an abundant Archaeal phylum that plays a major role in the global nitrogen cycle, specifically via the oxidation of ammonia. Environmental pH is a major factor affecting the distribution of different Thaumarchaeota clades (Gubry-Rangin et al. 2011). Phylogenetic methods could show that a radiation occurred early in the evolution of the Thaumarchaeota, allowing niche expansion from neutral pH environments to acidic and alkaline environments (Gubry-Rangin et al. 2015). Interestingly, diversification rate remained high after this initial burst, which is not consistent with typical adaptive radiations, where an initial high diversification is followed by a slowdown (a signature also observed in adaptive radiations inferred in bacteria (Morlon et al. 2012)). pH adaptation in Thaumarchaeota is at least in part mediated by V-type ATPase proton pumps (Wang et al. 2019). The phylogeny of acidophile V-type-like ATPase operons in Thaumarchaeota is incongruent with organismal phylogeny but is congruent with habitat, indicating that HGT is responsible for ATPase-mediated niche adaptation (Wang et al. 2019).

Ecological opportunity for adaptive radiations can be provided by abiotic factors such as resource type or pH as in the case studies above. But as prokaryotes are generally embedded in highly diverse and dense communities of competitors, parasites, prey, predators, hosts, symbionts, and mutualists, biotic factors must be highly relevant too. As different organisms can coevolve with each other, selection exerted by other organisms is not only likely to be strong, but also long lasting and potentially diversifying (Van Valen 1973). A meta-analysis on 16S diversity collected across many different biomes found that the diversity of specific lineages correlated positively with whole-community diversity (Madi et al. 2020). This observation is consistent with more diverse communities offering more available niche space through more diverse biotic interactions. It could also be shown that this relationship was weaker for the most diverse communities, indicating that when niches are increasingly filled, there is less opportunity for invading lineages to diversify (Madi et al. 2020).

## Entry into novel environments: adaptive zones

High dispersal rates mean that available niches are generally filled by the appropriate niche specialists. However, it also means that there is frequent immigration of taxa that are not (well) adapted to the local environment. The vast majority of such immigrants are unlikely to persist, let alone diversify (Madi et al. 2020). However, if an ecologically and genomically distinct migrant manages to take up a niche-defining gene from the local community, it could be in a position to occupy (or create) hitherto unexploited niche space and give rise to an adaptive radiation. An example of one of the most drastic environmental transitions for metazoans and prokaryotes alike is that between marine and terrestrial (including freshwater) environments (Cohan and Koeppel 2008, Logares et al. 2009). Salinity is a major determinant structuring microbial diversity, with distinct phylogenetic shifts observed over salt gradients (Dupont et al. 2014, Fortunato and Crump 2015). Successful marine–terrestrial transitions require significant rewiring of central metabolism and osmotic stress responses (Eiler et al. 2016), which could be aided by HGT (Wisniewski-Dye et al. 2011). Phylogenetic analyses indicate marine–terrestrial transitions occasionally occur in bacterial taxa (Zhang et al. 2019) and it can be argued these form an excellent model for the colonization of novel adaptive zones (Jurdzinski et al. 2023).

Another example of the colonization of novel adaptive zones is offered by pathogens switching host. *Staphylococcus aureus* infects a wide range of vertebrates (and even invertebrates) (Matuszewska et al. 2020). Host jumps are frequent and result in distinct genetic clusters where strains carry specific host-adaptive genes, and evidence loss of host-adaptive genes associated with their previous host (Matuszewska et al. 2020). Specifically, different host specialists are characterized by the carriage of different combinations of mobile genetic elements, including genes known to target specific host innate immune responses and antimicrobial resistance genes conferring resistance to antibiotics used in particular husbandry regimes (Richardson et al. 2018, Matuszewska et al. 2020). This further exemplifies the pervasive role of HGT in opening up new niches, although it is not clear whether MGEs are generally acquired just before or after host-switching events (Richardson et al. 2018).

Major new microbial niches have originated throughout Earth’s history, from the emergence of oxygenic habitats allowing aerobic respiration to the evolution of animal and plant hosts (Jaffe et al. 2023). Such niches range from ‘closed’ with purely vertical transmission (as those occupied by endosymbionts) to ‘open’ with mainly horizontal transmission (as those occupied by planktonic marine bacteria). Dispersal needs to occur to allow the colonization of novel adaptive zones, but it is not clear whether migration rates must be very high to allow rare key innovations to occur, or if they need to be at some intermediate level to prevent establishment of the best currently adapted species, in turn preventing the opportunity of a new best-adapted lineage to evolve.

## Generalists as progenitors of adaptive radiations

Prokaryotes can be classified as specialists or generalists based on the broadness of their niche requirements (Bell and Bell 2021). Bacteria with larger genomes and higher metabolic versatility are associated with greater niche width (Barberan et al. 2014). Living in a wider range of microbiomes means that such generalist species will encounter more distinct selection pressures as well as interact with more species that could serve as donors of key adaptations through HGT. A large-scale meta-analysis of 16S sequence data found that 16S OTUs present across a greater number of distinct habitats (likely to be generalists) was found to have a 19-fold higher speciation rate than OTUs present in only a single habitat (likely to be specialists) (Sriswasdi et al. 2017). That generalist-to-specialist transitions are more common than *vice versa*, is consistent with increasing specialization resulting in the closing of doors leading to other ecological lifestyles, which is consistent with results from lab experiments on bacteria (Buckling et al. 2003).

## Are some taxa inherently more prone to adaptively radiate?

Speciation rate is dependent on ecological opportunity, but also on the rate at which new niche-defining traits can arise. Taxa that are more evolvable (Díaz Arenas and Cooper 2013), thus could be expected to be in a better position to radiate into novel types. Species-specific variation in factors such as mutation rate, generation time, and population size all influence the rate of adaptation to new niches, but a high frequency of HGT specifically can be expected to facilitate the evolution of key innovations (Lawrence 2001).

High rates of HGT mediated by gene transfer agents (GTAs; exapted bacteriophages that function to secrete host DNA) have been implicated in a well-documented case of a bacterial adaptive radiation (Guy et al. 2013). *Bartonella* are vectorborne, intracellular pathogens of mammals comprising multiple species-level clades. Two clades with similar host range display evidence of increased diversification, and both could be shown to have independently taken up the VirB type IV secretion system (T4SS), which acts to inject virulence factors into host cells (Engel et al. 2011). All ancestral strains harboured a GTA capable of *in vitro* gene transfer (Guy et al. 2013); interestingly, the GTA is colocated with the T4SS genes, which results in a higher-than-average chance of being secreted and taken up by other cells (Tamarit et al. 2018). It has been proposed that his coupling of niche-defining genes and genes increasing recombination has allowed the successful diversification of this pathogen genus (Guy et al. 2013).

It is important to stress that HGT transfers do not necessarily lead to adaptive radiations when they do not increase functional diversity or when ecological opportunity is absent. For instance, hybridization events where donor DNA replaces up to 20% of the recipient genome have been observed in a variety of human pathogens (Chen et al. 2014, Croucher and Klugman 2014) without concomitant diversification. Moreover, it is possible that high rates of HGT could impede, rather than promote adaptive radiations. One of the very few studies that has discussed the concept of key adaptations in the context of prokaryotes has argued that HGT hinders adaptive radiations, because it could result in key adaptations being transferred to many lineages rather than just a single one (Martin et al. 2004).

## Adaptive radiations with and without speciation: implications for pangenome evolution

**Figure 3.**
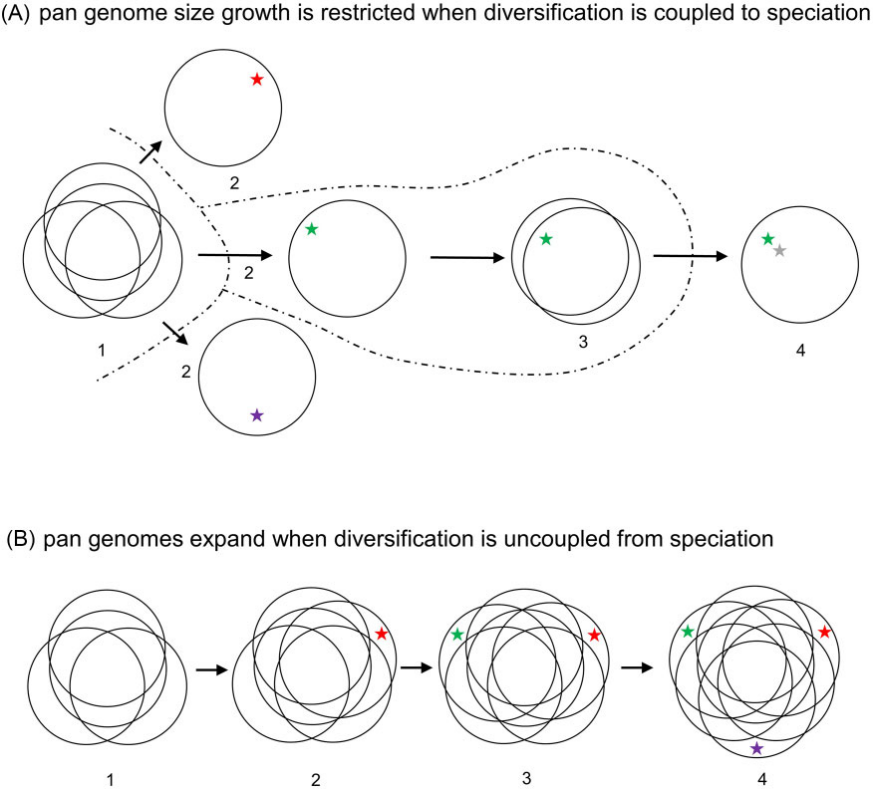
Pangenome diversification with and without barriers to recombination. (A) Diversification coupled to speciation in a species with barriers to recombination. The ancestral pangenome (1) acquires different key innovations (2); each uniquely adapted lineage ceases to recombine with the ancestor or with other newly evolved niche specialists because of recombination barriers (dashed lines). New niche specialists subsequently grow their pangenome through adaptive and nonadaptive processes (3). When a new key innovation occurs (4), the process is repeated. (B) Diversification of a species without barriers to recombination. The ancestral pangenome (1) grows progressively with each key innovation, depicted by red (2), green (3), and purple (4) stars as well as nonadaptive gene additions.

HGT in bacteria, like meiotic sex in eukaryotes, is a double-edged sword: on one hand it is central to creating genetic diversity, but on the other hand it can impede genetic divergence of nascent niche specialists. Without some ecological or genetic barrier to HGT, diversification cannot proceed to the species-level (Shapiro and Polz 2014). It is possible that many adaptive radiations in prokaryotes are ‘stuck’ on the strain-level because there are no barriers to recombination allowing speciation to occur (Fig. 2D) (Shapiro and Polz 2014). As a consequence, there could be unappreciated links between ecological diversification, recombination barriers, and the evolution of pangenomes (Fig. 3).

The evolution and ecology of pangenomes, the total complement of genes within a species, which is usually much larger than the number of genes in any individual genome, is a topic of great interest in evolutionary microbiology (Bobay 2020, Domingo-Sananes and McInerney 2021). Several distinct, nonmutually exclusive hypotheses have been put forward to explain the existence of pangenomes. Some explanations invoke adaptive benefits where different gene repertoires correspond to differential niche specialization (Domingo-Sananes and McInerney 2021). Other explanations invoke neutral processes; some species might be more prone to take up genes by HGT because their genomes are more accommodating to novel genetic diversity or because they are surrounded by a higher diversity of community members (Brockhurst et al. 2019). Greater effective population size is expected to result in greater pangenome diversity (Andreani et al. 2017), specifically via retainment of accessory genes with near-neutral fitness effects (Bobay and Ochman 2018).

However, there is another potential explanation of why pangenome size can vary among species, which is directly linked to diversification. Every time a new niche specialist evolves and recombination with the ancestor ceases, the niche specialists start with a ‘minimal’ pangenome (Fig. 3A). Although this pangenome will increase in size during the lifetime of a species through adaptive processes (e.g. diversifying selection), nonadaptive processes (e.g. the uptake of parasitic mobile genetic elements) as well as neutral processes, it will be small initially. In contrast, if recombination barriers are absent, for instance when different genotypes remain in close physical contact, new niche specialists still evolve, but their core genes will remain tied together through continued recombination (Shapiro and Polz 2014). Clade-specific accessory genes will remain part of the pangenome, which will grow with the evolution of each new niche specialist (Fig. 3B). *Escherichia* might fit this latter scenario: species numbers in this genus are low and *E. coli* has a famously large pangenome. In this scenario, *E. coli* displays an evolutionary ‘shallow’ adaptive radiation where niche specialists are unable to evolve into species (Fig. 2D).

## Discussion and conclusions

Adaptive radiations have been implicated in bursts of species richness in animals and plants, and multiple high quality case studies have demonstrated that they also occur in bacteria. However, the study of adaptive radiations in prokaryotes is still in its infancy and many questions remain to be answered. For instance, are certain genetic (e.g. restriction/modification systems) or ecological characteristics (e.g. type of metabolism or microbiome) especially conducive to the radiation of lineages? Are particular traits unlikely to give rise to adaptive radiations because they are especially prone to horizontal spread and unlikely to transfer to a single lineage? Do key adaptations come as single genes or operons or can they be more complex, involving many genes, such as in the evolution of cell walls (Cohan and Koeppel 2008)? Could some radiations be started by purely mutational processes rather than HGT, as has been shown experimentally with the mutational evolution of citrate metabolism in *E. coli* (Blount et al. 2008)? Are some clades species-rich because they are old rather than having undergone burst-like evolution?

Generalization of patterns and processes between very different organisms and lifestyles is a main challenge (Gillespie et al. 2020). We would argue that bacterial diversification does not differ qualitatively from that in macrobes but only quantitatively. In other words: ‘prokaryotes also disperse, adapt, recombine and speciate, just to different extents’. HGT allows the uptake of complete operons from different species and could increase the likelihood of key innovations. This effect is likely much more pervasive but not wholly different from hybridization events preceding adaptive radiations in eukaryote species (Seehausen 2004). When genome-wide HGT remains ongoing between differentially adapted lineages this means that adaptive radiations cannot proceed and will not result in increased species richness, but rather highly diverse ‘strain flocks’. The same process has been observed in sticklebacks, where speciation also occurs along a continuum, including repeated and reversible specialization and reproductive isolation (Hendry et al. 2009). Arguably the most pronounced difference between prokaryotes and multicellular organisms is that environmental filtering is much more important than dispersal limitation.

In-depth genomic and ecological knowledge on species and ecotypes will be necessary to identify patterns of increased diversification, links to ecological niches, barriers to recombination and specific key innovations. As in all fields of microbiology, the way we study bacterial diversification depends greatly on technological advances. Increasing sequencing power allows for the routine use of metagenome assembled genomes (Parks et al. 2017). Ancient DNA (Wibowo et al. 2021), HGT transfers (Davín et al. 2018), and bacteria–eukaryote associations (Wang and Luo 2021) all can help explicitly date radiations and aid in the reconstruction of ancestral states. Despite technical and computational challenges, it could be argued it is actually easier to study adaptive radiations in bacteria, as vicariance is less important relative to selection. In addition, genomic diversification is more tractable compared to macrobes and experiments can be designed to test the ecological function of genes under controlled lab conditions. Experimental evolution studies incorporating multiple species and allowing HGT (e.g. Hall et al. 2016) are a crucial way forward to study diversification. We look forward to more high-resolution genomic studies of natural populations examining the interplay between ecology, evolution, and genetics that ultimately leads to diversification of clades, genomes, pangenomes, and microbial communities.

## References

[bib1] Alfaro ME . Key evolutionary innovations. In: Baum DA, Futuyma DJ, Hoekstra HE, Lenski RE, Moore AJ, Peichel CL, Schluter D, Whitlock MC (eds), The Princeton Guide to Evolution. Princeton: Princeton University Press, 2014, 594–600.

[bib2] Andreani NA , Hesse E, Vos M. Prokaryote genome fluidity is dependent on effective population size. ISME J. 2017;11:1719–21. 28362722 10.1038/ismej.2017.36 PMC5520154

[bib3] Baas Becking LGM . Geobiologie of Inleiding Tot De Milieukunde. Den Haag: WP Van Stockum & Zoon NV, 1934.

[bib4] Bahram M , Hildebrand F, Forslund SK et al. Structure and function of the global topsoil microbiome. Nature. 2018;560:233–7. 30069051 10.1038/s41586-018-0386-6

[bib79_358_274023] Barberán A. , Ramirez KS, Leff JW et al. Why are some microbes more ubiquitous than others? Predicting the habitat breadth of soil bacteria. Ecol Lett. 2014;17:794–802. 10.1111/ele.12282 24751288

[bib6] Bell TH , Bell T. Many roads to bacterial generalism. FEMS Microbiol Ecol. 2021;97:fiaa240. 10.1093/femsec/fiaa240 33238305

[bib7] Blount ZD , Borland CZ, Lenski RE. Historical contingency and the evolution of a key innovation in an experimental population of Escherichia coli. Proc Natl Acad Sci USA. 2008;105:7899–906. 18524956 10.1073/pnas.0803151105 PMC2430337

[bib8] Bobay L-M , Ochman H. Factors driving effective population size and pan-genome evolution in bacteria. BMC Evol Biol. 2018;18:1–12. 30314447 10.1186/s12862-018-1272-4 PMC6186134

[bib9] Bobay L-M . The prokaryotic species concept and challenges. The Pangenome. 2020;25:21–49. 32633914

[bib10] Brockhurst MA , Harrison E, Hall JP et al. The ecology and evolution of pangenomes. Curr Biol. 2019;29:R1094–R103. 31639358 10.1016/j.cub.2019.08.012

[bib11] Buckling A , Wills MA, Colegrave N. Adaptation limits diversification of experimental bacterial populations. Science. 2003;302:2107–9. 14684817 10.1126/science.1088848

[bib12] Chen L , Mathema B, Pitout JD et al. Epidemic *Klebsiella pneumoniae* ST258 is a hybrid strain. mBio. 2014;5:e01355–14. 24961694 10.1128/mBio.01355-14 PMC4073492

[bib13] Cohan FM , Koeppel AF. The origins of ecological diversity in prokaryotes. Curr Biol. 2008;18:R1024–R34. 19000803 10.1016/j.cub.2008.09.014

[bib14] Croucher NJ , Klugman KP. The emergence of bacterial “hopeful monsters”. mBio. 2014;5:e01550–14. 25073645 10.1128/mBio.01550-14 PMC4128365

[bib15] Davín AA , Tannier E, Williams TA et al. Gene transfers can date the tree of life. Nat Ecol Evol. 2018;2:904–9. 29610471 10.1038/s41559-018-0525-3 PMC5912509

[bib16] Díaz Arenas C , Cooper TF. Mechanisms and selection of evolvability: experimental evidence. FEMS Microbiol Rev. 2013;37:572–82. 23078278 10.1111/1574-6976.12008

[bib17] Domingo-Sananes MR , McInerney JO. Mechanisms that shape microbial pangenomes. Trends Microbiol. 2021; 29: 493–503. 33423895 10.1016/j.tim.2020.12.004

[bib18] Dupont CL , Larsson J, Yooseph S et al. Functional tradeoffs underpin salinity-driven divergence in microbial community composition. PLoS ONE. 2014;9:e89549. 24586863 10.1371/journal.pone.0089549 PMC3937345

[bib19] Dykhuizen DE . Santa Rosalia revisited: why are there so many species of bacteria?. Antonie Van Leeuwenhoek. 1998;73:25–33. 9602276 10.1023/a:1000665216662

[bib20] Eiler A , Mondav R, Sinclair L et al. Tuning fresh: radiation through rewiring of central metabolism in streamlined bacteria. ISME J. 2016;10:1902. 26784354 10.1038/ismej.2015.260 PMC5029164

[bib21] Engel P , Salzburger W, Liesch M et al. Parallel evolution of a type IV secretion system in radiating lineages of the host-restricted bacterial pathogen Bartonella. PLoS Genet. 2011;7:e1001296. 21347280 10.1371/journal.pgen.1001296 PMC3037411

[bib22] Fortunato CS , Crump BC. Microbial gene abundance and expression patterns across a river to ocean salinity gradient. PLoS ONE. 2015;10:e0140578. 26536246 10.1371/journal.pone.0140578 PMC4633275

[bib23] Gavrilets S , Losos JB. Adaptive radiation: contrasting theory with data. Science. 2009;323:732–7. 19197052 10.1126/science.1157966

[bib24] Gillespie RG , Bennett GM, De Meester L et al. Comparing adaptive radiations across space, time, and taxa. J Hered. 2020;111:1–20. 31958131 10.1093/jhered/esz064 PMC7931853

[bib25] Gubry-Rangin C , Hai B, Quince C et al. Niche specialization of terrestrial archaeal ammonia oxidizers. Proc Natl Acad Sci USA. 2011;108:21206–11. 22158986 10.1073/pnas.1109000108 PMC3248517

[bib26] Gubry-Rangin C , Kratsch C, Williams TA et al. Coupling of diversification and pH adaptation during the evolution of terrestrial thaumarchaeota. Proc Natl Acad Sci USA. 2015;112:9370–5. 26170282 10.1073/pnas.1419329112 PMC4522744

[bib27] Guy L , Nystedt B, Toft C et al. A gene transfer agent and a dynamic repertoire of secretion systems hold the keys to the explosive radiation of the emerging pathogen Bartonella. PLoS Genet. 2013;9:e1003393. 23555299 10.1371/journal.pgen.1003393 PMC3610622

[bib28] Hall JP , Brockhurst MA, Harrison E. Sampling the mobile gene pool: innovation via horizontal gene transfer in bacteria. Philos Trans R Soc Lond B Biol Sci. 2017;372:20160424. 29061896 10.1098/rstb.2016.0424 PMC5665811

[bib29] Hall JP , Wood AJ, Harrison E et al. Source–sink plasmid transfer dynamics maintain gene mobility in soil bacterial communities. Proc Natl Acad Sci USA. 2016;113:8260–5. 27385827 10.1073/pnas.1600974113 PMC4961173

[bib30] Heard SB , Hauser DL. Key evolutionary innovations and their ecological mechanisms. Hist Biol. 1995;10:151–73.

[bib31] Heath TA , Hedtke SM, Hillis DM. Taxon sampling and the accuracy of phylogenetic analyses. J Syst Evol. 2008;46:239–57.

[bib32] Hehemann J-H , Arevalo P, Datta MS et al. Adaptive radiation by waves of gene transfer leads to fine-scale resource partitioning in marine microbes. Nat Comms. 2016;7:12860. 10.1038/ncomms12860 PMC5036157 27653556

[bib33] Hendry AP , Bolnick DI, Berner D et al. Along the speciation continuum in sticklebacks. J Fish Biol. 2009;75:2000–36. 20738669 10.1111/j.1095-8649.2009.02419.x

[bib34] Hunt DE , David LA, Gevers D et al. Resource partitioning and sympatric differentiation among closely related bacterioplankton. Science. 2008;320:1081–5. 18497299 10.1126/science.1157890

[bib35] Hunter JP . Key innovations and the ecology of macroevolution. Trends Ecol Evol. 1998;13:31–6. 21238187 10.1016/s0169-5347(97)01273-1

[bib36] Hutchinson GE . Homage to Santa Rosalia or why are there so many kinds of animals?. Am Nat. 1959;93:145–59.

[bib37] Jaffe AL , Castelle CJ, Banfield JF. Habitat transition in the evolution of bacteria and archaea. Annu Rev Microbiol. 2023;77:193–212. 37100405 10.1146/annurev-micro-041320-032304

[bib38] Jurdzinski KT , Mehrshad M, Delgado LF et al. Large-scale phylogenomics of aquatic bacteria reveal molecular mechanisms for adaptation to salinity. Sci Adv. 2023;9:eadg2059. 37235649 10.1126/sciadv.adg2059 PMC10219603

[bib39] Larsen BB , Miller EC, Rhodes MK et al. Inordinate fondness multiplied and redistributed: the number of species on earth and the new pie of life. Q Rev Biol. 2017;92:229–65.

[bib40] Lawrence JG . Catalyzing bacterial speciation: correlating lateral transfer with genetic headroom. Syst Biol. 2001;50:479–96. 12116648 10.1080/10635150120286

[bib41] Locey KJ , Lennon JT. Scaling laws predict global microbial diversity. Proc Natl Acad Sci USA. 2016;113:5970–5. 27140646 10.1073/pnas.1521291113 PMC4889364

[bib42] Logares R , Bråte J, Bertilsson S et al. Infrequent marine–freshwater transitions in the microbial world. Trends Microbiol. 2009;17:414–22. 19726194 10.1016/j.tim.2009.05.010

[bib43] Loren JG , Farfan M, Fuste MC. Molecular phylogenetics and temporal diversification in the genus *Aeromonas* based on the sequences of five housekeeping genes. PLoS ONE. 2014;9:e88805. 24586399 10.1371/journal.pone.0088805 PMC3930666

[bib44] Louca S , Mazel F, Doebeli M et al. A census-based estimate of Earth’s bacterial and archaeal diversity. Plos Biol. 2019;17:e3000106. 30716065 10.1371/journal.pbio.3000106 PMC6361415

[bib45] Louca S , Shih PM, Pennell MW et al. Bacterial diversification through geological time. Nat Ecol Evol. 2018;2:1458–67. 30061564 10.1038/s41559-018-0625-0

[bib46] Louca S . The rates of global bacterial and archaeal dispersal. ISME J. 2022;16:159–67. 34282284 10.1038/s41396-021-01069-8 PMC8692594

[bib47] Lozupone C , Knight R. UniFrac: a new phylogenetic method for comparing microbial communities. Appl Env Microbiol. 2005;71:8228–35. 16332807 10.1128/AEM.71.12.8228-8235.2005 PMC1317376

[bib48] MacLean CR . Adaptive radiation in microbial microcosms. J Evol Biol. 2005;18:1376–86. 16313450 10.1111/j.1420-9101.2005.00931.x

[bib49] Madi N , Vos M, Legendre P et al. Diversity begets diversity in microbiomes. eLife. 2020;9:e58999. 33215610 10.7554/eLife.58999 PMC7755399

[bib50] Marin J , Battistuzzi FU, Brown AC et al. The timetree of prokaryotes: new insights into their evolution and speciation. Mol Biol Evol. 2016;34:437–46. 10.1093/molbev/msw245 27965376

[bib51] Martin AP , Costello EK, Meyer AF et al. The rate and pattern of cladogenesis in microbes. Evolution. 2004;58:946–55. 15212376 10.1111/j.0014-3820.2004.tb00429.x

[bib52] Matschiner M , Colombo M, Damerau M et al. The Adaptive Radiation of Notothenioid Fishes in the Waters of Antarctica Extremophile Fishes. Berlin: Springer, 2015, 35–57.

[bib53] Matuszewska M , Murray GG, Harrison EM et al. The evolutionary genomics of host specificity in *Staphylococcus aureus*. Trends Microbiol. 2020;28:465–77. 31948727 10.1016/j.tim.2019.12.007

[bib54] Mooers AO , Heard SB. Inferring evolutionary process from phylogenetic tree shape. Q Rev Biol. 1997;72:31–54.

[bib55] Mora C , Tittensor DP, Adl S et al. How many species are there on Earth and in the ocean?. Plos Biol. 2011;9:e1001127. 21886479 10.1371/journal.pbio.1001127 PMC3160336

[bib56] Morlon H , Kemps BD, Plotkin JB et al. Explosive radiation of a bacterial species group. Evolution. 2012;66:2577–86. 22834754 10.1111/j.1558-5646.2012.01598.x PMC3871994

[bib57] O’Dwyer JP , Kembel SW, Sharpton TJ. Backbones of evolutionary history test biodiversity theory for microbes. Proc Natl Acad Sci USA. 2015;112:8356–61. 26106159 10.1073/pnas.1419341112 PMC4500224

[bib58] Parks DH , Chuvochina M, Waite DW et al. A standardized bacterial taxonomy based on genome phylogeny substantially revises the tree of life. Nat Biotechnol. 2018;36:996–1004. 30148503 10.1038/nbt.4229

[bib81_1701142113184] Parks DH , Rinke C, Chuvochina M et al. Recovery of nearly 8,000 metagenome-assembled genomes substantially expands the tree of life. Nat Microbiol. 2017;2:1533–42. 28894102 10.1038/s41564-017-0012-7

[bib59] Quince C , Curtis TP, Sloan WT. The rational exploration of microbial diversity. ISME J. 2008;2:997–1006. 18650928 10.1038/ismej.2008.69

[bib78_862_272823] Rainey PB , Travisano M. Adaptive radiation in a heterogeneous environment. Nature. 1998;394:69–72. 9665128 10.1038/27900

[bib60] Ramirez KS , Leff JW, Barberán A et al. Biogeographic patterns in below-ground diversity in New York City’s Central Park are similar to those observed globally. Proc R Soc B Biol Sci. 2014;281:20141988. 10.1098/rspb.2014.1988 PMC4213626 25274366

[bib61] Richardson EJ , Bacigalupe R, Harrison EM et al. Gene exchange drives the ecological success of a multi-host bacterial pathogen. Nat Ecol Evol. 2018;2:1468–78. 30038246 10.1038/s41559-018-0617-0 PMC7610605

[bib62] Rosenzweig ML . Species Diversity in Space and Time. Cambridge: Cambridge University Press, 1995.

[bib63] Schluter D . The Ecology of Adaptive Radiation. Oxford: Oxford University Press, 2000.

[bib64] Scholl JP , Wiens JJ. Diversification rates and species richness across the Tree of Life. Proc R Soc B Biol Sci. 2016;283:20161334. 10.1098/rspb.2016.1334 PMC5031659 27605507

[bib65] Seehausen O . Hybridization and adaptive radiation. Trends Ecol Evol. 2004;19:198–207. 16701254 10.1016/j.tree.2004.01.003

[bib67] Shapiro BJ , Polz MF. Ordering microbial diversity into ecologically and genetically cohesive units. Trends Microbiol. 2014;22:235–47. 24630527 10.1016/j.tim.2014.02.006 PMC4103024

[bib68] Sriswasdi S , Yang C-c, Iwasaki W. Generalist species drive microbial dispersion and evolution. Nat Comms. 2017;8:1–8. 10.1038/s41467-017-01265-1 PMC5660117 29079803

[bib69] Tamarit D , Neuvonen M-M, Engel P et al. Origin and evolution of the *Bartonella* gene transfer agent. Mol Biol Evol. 2018;35:451–64. 29161442 10.1093/molbev/msx299

[bib70] Travisano M , Rainey PB. Studies of adaptive radiation using model microbial systems. Am Nat. 2000;156:S35–44. 29592580 10.1086/303414

[bib71] Van Valen L . A new evolutionary law. Evol Theory. 1973;1:1–30.

[bib72] Vos M . A species concept for bacteria based on adaptive divergence. Trends Microbiol. 2011;19:1–7. 21071229 10.1016/j.tim.2010.10.003

[bib73] Wang B , Qin W, Ren Y et al. Expansion of Thaumarchaeota habitat range is correlated with horizontal transfer of ATPase operons. ISME J. 2019;13:3067–79. 31462715 10.1038/s41396-019-0493-x PMC6863869

[bib74] Wang S , Luo H. Dating Alphaproteobacteria evolution with eukaryotic fossils. Nat Comms. 2021;12:1–9. 10.1038/s41467-021-23645-4 PMC8175736 34083540

[bib80_130_274523] Whitaker RJ , Grogan DW, Taylor JW. Geographic barriers isolate endemic populations of hyperthermophilic archaea. Science. 2003;301:976–8. 12881573 10.1126/science.1086909

[bib75] Wibowo MC , Yang Z, Borry M et al. Reconstruction of ancient microbial genomes from the human gut. Nature. 2021;594:234–9. 33981035 10.1038/s41586-021-03532-0 PMC8189908

[bib76] Wisniewski-Dye F , Borziak K, Khalsa-Moyers G et al. *Azospirillum* genomes reveal transition of bacteria from aquatic to terrestrial environments. PLoS Genet. 2011;7:e1002430. 22216014 10.1371/journal.pgen.1002430 PMC3245306

[bib77] Yarza P , Yilmaz P, Pruesse E et al. Uniting the classification of cultured and uncultured bacteria and archaea using 16S rRNA gene sequences. Nat Rev Microbiol. 2014;12:635–45. 25118885 10.1038/nrmicro3330

[bib78] Zhang H , Yoshizawa S, Sun Y et al. Repeated evolutionary transitions of flavobacteria from marine to non-marine habitats. Env Microbiol. 2019;21:648–66. 30565818 10.1111/1462-2920.14509

